# Should trainee doctors use the developing world to gain clinical experience? The annual Varsity Medical Debate – London, Friday 20^th^ January, 2012

**DOI:** 10.1186/1747-5341-8-1

**Published:** 2013-02-21

**Authors:** Barnabas J Gilbert, Calum Miller, Fenella Corrick, Robert A Watson

**Affiliations:** 1 Green Templeton College, Woodstock Road, Oxford, OX2 6HG, UK; 2 St Hugh’s College, St Margaret’s Road, Oxford, OX2 6LE, UK; 3 Oriel College, Oxford, OX1 4EW, UK

**Keywords:** Elective, Education, Clinical experience, Overseas, Developing world

## Abstract

The 2012 Varsity Medical Debate between Oxford University and Cambridge University provided a stage for representatives from these famous institutions to debate the motion “This house believes that trainee doctors should be able to use the developing world to gain clinical experience.” This article brings together many of the arguments put forward during the debate, centring around three major points of contention: the potential intrinsic wrong of ‘using’ patients in developing countries; the effects on the elective participant; and the effects on the host community. The article goes on to critically appraise overseas elective programmes, offering a number of solutions that would help optimise their effectiveness in the developing world.

## Introduction

Since the 1970s, medical students at most western medical schools and many clinicians and surgeons completing specialty training have undertaken medical ‘electives’ [1,2]. Such schemes typically involve 6–8 week periods in which participants ‘visit a community with cultural and societal norms different from those of their home’ [3]. This article pertains primarily to the 40% of medical electives which take place in developing countries [4]. Given that 3000 of the 8000 doctors produced by the UK each year spend their elective in a developing country, this equates to over 350 aggregate years invested in these communities per annum [5]. Theoretically, the benefits to the trainee and the host community are rich. However, with a scarcity of empirical confirmation, and in light of the declining life expectancy and rising healthcare costs seen in many developing countries [6,7], it is surely time to establish precisely what is achieved by the traditional medical elective.

### The intrinsic wrong of ‘use’

The General Medical Council requires that all medical students and doctors ‘act with integrity’ and ‘work within their limits of competence’ [8]. The British Medical Journal adds that it is wrong for trainees to take on the role of a qualified doctor ‘irrespective of any encouragement they may receive from the host organisations to which they are attached’ [9]. This stance suggests that elective students should approach foreign patients just as they would approach patients in their own country. They should avoid ‘practising on the poor’ [10] on the grounds that it is exploitative and morally unjustifiable. Given that the most frequent expectation of medical residents (94%) is the acquisition of clinical skills [11], this apparent conflict ought to be taken seriously.

Any fear of an intrinsic wrong in the ‘use’ of developing countries might be alleviated by noting that medical education in *all* countries is fundamentally structured around students ‘using’ patients. However, the ‘use’ of desperate patients who have little choice other than to accept elective students’ intervention may constitute an ethically problematic ‘use’ which is unique to overseas electives. Moreover, the greater extent to which students perform procedures beyond their competence during electives in developing countries may plausibly make the ‘use’ of patients more troubling in these contexts.

Whilst some suggest that medical trainees are morally obliged to help those who would otherwise have no access to medical care [12], it could be argued that this attitude of utility “contributes to the ethos of the global health inequity” [2]. This assumption that the rights of the poor differ from the rights of the wealthy may influence the broader socio-political discourse and the way in which medical and political organisations tackle inequalities in the long term.

### Effects on the elective participant

Despite a paucity of pertinent literature, with available evidence tending to rely on self-reported questionnaire accounts, evaluation of existing surveys reveals several advantages of the elective programme for the partaking trainee. Firstly, electives offer trainee doctors the opportunity to develop their clinical skills. In an environment often void of advanced medical technology, there is added emphasis on diagnostic reasoning and history-taking, skills perceived by some to receive insufficient attention in western curricula [13]. Trainees encounter novel diseases, expanding the range of their clinical skill set. If migrants to the West carry these diseases, trainees may find themselves uniquely placed to manage these conditions later in their careers [14]. Secondly, experience in the developing world cultivates interest in, and heightens the profile of, global health issues. A study by Haq et al. (2000) reported major attitudinal changes in 83% of post-elective trainees, with 73% of respondents participating in a further international health scheme and 27% pursuing graduate studies in public health [15].

Although some elective programmes have rigorous selection criteria and well-established supervision, trainees may find themselves attached to resource-depleted hospitals. Alternatively, they may be left to ‘do their best’ to manage heavy workloads with limited or no supervision [10], leading to the acquisition of poor practice habits. As many as 40% of elective participants admit being asked to act outside their competence [16], often with emotional and hierarchical pressure to do so. Twelve trainees interviewed in a survey by Elit et al. (2011) noted a ‘see one, do one, teach one’ approach applied to procedures including lumbar puncture and obstetrical deliveries [17]. One respondent reported how he had counselled an HIV positive patient without training or supervision. These examples illustrate the potential for a participant to inaccurately appraise his or her own competence and relative ability to practice safely. This practice is difficult to prevent, however, since participants who remain within their level of expertise often find their limited ability to intervene demoralising [17].

### Effects on the host community

Although evaluation of the impact of elective schemes on host communities has been sparse, it appears that a non-negligible proportion of the worldwide elective programme include elements of ‘medical tourism’, where the host community is disadvantaged by the trainee’s presence [16]. There are a number of means by which such a scheme could damage the local community. By providing temporary solutions, they may undermine local healthcare infrastructure [18] or by functioning as a duplicate service, they may divert patients who have the ability to pay away from the existing local healthcare system. Local healthcare professionals may find themselves unable to compete with a free volunteer service [19], with the neediest patients prevented from receiving treatment.

These risks are not universally realised, however; it is important to acknowledge that long-term reciprocal relationships between institutions may be to the benefit of the host institution. In particular, long-term partnerships between educational institutions in developed and developing countries should be encouraged, as exemplified by the link between several western medical schools and Moi University in Kenya [20]. Such relationships provide the host country with a predictable supply of elective students at a standardised level of training and also offer local trainees opportunities for electives in association with western medical schools.

An incompetent trainee can hinder proper medical care by providing false information, performing dangerous or technically demanding procedures without requisite expertise, or by spreading infections throughout a host community [18]. However, many tasks allocated to doctors in the developed world are performed in the developing world by other members of the healthcare team. These staff may lack formal training but learn to perform these tasks, often to an exceptionally high standard. On the one hand, this suggests that the roles of professionals can be performed by elective trainees who lack formal training, but at the same time it is unclear whether electives are long enough for this expertise to develop sufficiently. Although local healthcare workers may be available who could do a better job using available resources, patients may prefer to be treated by a western trainee, and may willingly undergo riskier procedures than they would otherwise allow a local physician to perform [21,22]. Notably, most host country staff questioned in a survey by Radstone et al. (2005) thought trainee doctors should be able to diagnose (94.9%), prescribe (84.6%) and treat patients (89.7%) without supervision, but were unaware that this was forbidden in the trainee’s home country [10].

Trainees may be unable to discern the line between ‘advocating for the patient’ and ‘fitting in with the team’ [17]. The language barrier may heighten such tension. However, communicative difficulties should not detract from, but rather encourage participation in electives, as many trainees will return to communities where an ability to manoeuvre and transcend the language barrier is essential. In seeking to change medical practice in the host community, trainees may be cautious of adopting a paternalistic approach, instead preferring to model change through simple hygienic and communicative measures [17].

### Solutions

Many of these issues conflict with the recommendation of the Crisp Report on Global Health Partnerships [6] that ‘healthcare schools should work with others to ensure… training placements in developing countries are beneficial to the receiving country’. However, efforts are being made to reduce patient harm through pre-departure training schemes, as exemplified by the University of Toronto [23], which offer departing elective trainees the opportunity to discuss hypothetical case studies of ethical dilemmas they may face.

In the future, more elective programmes should seek long-term, sustainable relationships which promote accountability, exchange of ideas and collaborative agreement between all parties, in order to ensure consistency of practice and the achievement of community goals. The development of such programmes requires rigorous regulation and long-term planning, but successes such as the relationship between Australia’s sending institutions and India’s host communities [24] demonstrate the viability of this approach.

In order to transform a process favouring the trainee into an equitable exchange, each trainee must recognise the need for reciprocity when a community contributes to his or her education. This might manifest through the provision of resources, such as books and surgical supplies, of teaching and new ideas, or of money, which could be reallocated to meet local need.

The establishment of a central record of elective participants from all sending institutions could facilitate discussions between previous participants, forming a platform on which to introduce the social and cultural context of each trip and to discuss lessons learned. Where possible, elective duration should be maximised and rolling programmes set up to provide a continual stream of trainees to the neediest regions, indicative of a long-term care commitment to the host.

Contractual agreements should be drawn up to help meet pre-established criteria for professional supervision. Each contract should inform the local healthcare team of the role of the elective student in their home country, such that informed decisions can be made about the level of responsibility to warrant each trainee. The administrative costs of such a programme could be reduced by appointing and training one healthcare professional within each partner institution to oversee these amendments and to provide an obvious channel for raising concern.

Both the number of patients treated and the quality of patient care could be optimised by allowing trainees to hone a narrowed set of procedural skills within a chosen specialty, as has been a success in the case of ophthalmological surgical electives [25]. Accordingly, those planning medical school curricula should advocate the running of such programmes towards the end of a student’s clinical training. This should help trainees to avoid fitness to practice issues which may result if they exceed the bounds of their clinical competence.

## Conclusion

Electives should be able to refresh and invigorate a participant’s outlook on their future role as a doctor within the wider setting of global health, whilst simultaneously improving the health and social wellbeing of the host community. For this to happen, those organising such schemes must understand that the potential for patient harm cannot be ignored. With appropriate organisation and planning, trainee doctors could make a significant and sustainable difference. This may best take place within the context of interdisciplinary intervention, seeking to maximise community cohesion through a more holistic approach to patient care.

### About the debate

The Varsity Medical Debate was started in 2008 with the aim of creating a discourse on ethical issues among medical students. Embracing the historical rivalry between the two Universities, the debate encourages medics from Oxford and Cambridge to consider and articulate arguments surrounding issues that will feature heavily in their future careers. The debate is judged on the grounds of logic, coherency and presentation.

The 2012 Varsity Medical Debate took place at the Royal College of General Practitioners on January 20^th^. The judges narrowly awarded this year’s victory to the Cambridge proposition.

## Competing interests

The authors declare that they have no competing interests.

## Authors’ contributions

All authors planned and elucidated the layout. BJG drafted the manuscript which was critically edited and added to by CM, FC and RAW. All authors have read and approved the final draft.
